# The Cellular Robustness by Genetic Redundancy in Budding Yeast

**DOI:** 10.1371/journal.pgen.1001187

**Published:** 2010-11-04

**Authors:** Jingjing Li, Zineng Yuan, Zhaolei Zhang

**Affiliations:** 1 Department of Molecular Genetics, University of Toronto, Toronto, Canada; 2 Donnelly Centre for Cellular and Biomolecular Research, University of Toronto, Toronto, Canada; 3 Banting and Best Department of Medical Research, University of Toronto, Toronto, Canada; University of Michigan, United States of America

## Abstract

The frequent dispensability of duplicated genes in budding yeast is heralded as a hallmark of genetic robustness contributed by genetic redundancy. However, theoretical predictions suggest such backup by redundancy is evolutionarily unstable, and the extent of genetic robustness contributed from redundancy remains controversial. It is anticipated that, to achieve mutual buffering, the duplicated paralogs must at least share some functional overlap. However, counter-intuitively, several recent studies reported little functional redundancy between these buffering duplicates. The large yeast genetic interactions released recently allowed us to address these issues on a genome-wide scale. We herein characterized the synthetic genetic interactions for ∼500 pairs of yeast duplicated genes originated from either whole-genome duplication (WGD) or small-scale duplication (SSD) events. We established that functional redundancy between duplicates is a pre-requisite and thus is highly predictive of their backup capacity. This observation was particularly pronounced with the use of a newly introduced metric in scoring functional overlap between paralogs on the basis of gene ontology annotations. Even though mutual buffering was observed to be prevalent among duplicated genes, we showed that the observed backup capacity is largely an evolutionarily transient state. The loss of backup capacity generally follows a neutral mode, with the buffering strength decreasing in proportion to divergence time, and the vast majority of the paralogs have already lost their backup capacity. These observations validated previous theoretic predictions about instability of genetic redundancy. However, departing from the general neutral mode, intriguingly, our analysis revealed the presence of natural selection in stabilizing functional overlap between SSD pairs. These selected pairs, both WGD and SSD, tend to have decelerated functional evolution, have higher propensities of co-clustering into the same protein complexes, and share common interacting partners. Our study revealed the general principles for the long-term retention of genetic redundancy.

## Author Summary

Eukaryotic cells show remarkable robustness against external perturbations, which has been thought to be attributed, at least in part, to the extensive gene duplication events in eukaryotic genomes. By duplication, genes are likely to gain redundant copies for backup purposes, however, this notion contradicts the population genetic theory that genetic redundancy is evolutionarily unstable. In this study, we used yeast as a model organism to delineate the evolutionary trajectory of genetic robustness by gene duplication, utilizing the comprehensively characterized synthetic genetic interaction data in the yeast genome. We showed that the evolution of genetic robustness by duplication follows a neutral mode, with the loss of backup capacity proportional to the divergence time. However, natural selection was also acting on a few pairs to maintain their long-term backup capacity; and these pairs are slowly evolving, are co-clustered in the same protein complexes, and tend to interact with the similar partners. This study unravels the general principles underlying the evolution of the cellular robustness arising from genetic redundancy.

## Introduction

Genetic robustness in yeast cells accounts for insignificant phenotypic consequences upon deletion of many genes [1], [2]. It is thought that such resilient design of the genetic program is achieved in two different ways. In the first scenario, genes performing related functions are distributed on alternate pathways [3], [4] mimicking the electric parallel circuits so its alternate paths can compensate that blockage of one pathway. The second strategy to achieve robustness is by gene duplication, *i.e.* null mutation on one gene can be buffered by its paralogous copy which shares overlapping function [5]. This notion is supported by recent investigations which showed that mutual compensation is prevalent among paralogs [6]–[8], but contradicts population genetic theories predicting that genetic redundancy is evolutionarily unstable [9]. The instability can be understood when considering the evolutionary fate of duplicated genes [10]. Upon duplication, the paralogs usually go through a short-lived and transient state of complete redundancy, followed by a non-functionalization process that leads to massive loss of duplicates [10]. To persist, duplicate genes usually have to functionally diverge, either through subfunctionalization (partition of ancestral functions) or neofunctionalization (independent gain of novel functions) [10]–[13]. Regardless of how the paralogs had navigated an evolutionary trajectory from the transient complete redundancy to the long-time retention, the sister paralogs are anticipated to share fewer functions as time progresses. Therefore, the missive loss of duplicated genes and the highly divergent functions between the long-term retained pairs appear to be contradictory to the genetic redundancy provided by paralogs. More perplexingly, even for the duplicates that have backup capacity, several recent studies reported that little functional similarity is shared between them [8], [14], leading to the hypothesis of “backup without redundancy” [14].

As these previous observations were made on small datasets from double-gene deletion experiments in budding yeast, it is necessary to re-examine the relationship between the cellular robustness and gene redundancy using more recent and larger datasets, and more importantly, to include paralogs arising from different evolutionary origins. In this study, we based our analysis on the synthetic genetic interactions derived from a recent landmark study, in which ∼2, 000 genes were queried against the rest of the genome for synthetic genetic interactions (epistasis) [15]. This data set, larger than any other previous yeast double-deletion experiments, provides us a unique opportunity to systematically examine the genetic buffering between ∼500 duplicate gene pairs on a genome-wide scale. Moreover, this data set includes duplicate pairs from both whole-genome duplication (WGD) and small-scale duplications (SSD), allowing us to compare duplicates with different origins in an unbiased manner. Our analysis confirmed the previous reports, which were based on much smaller datasets, about the prevalent mutual compensation among paralogs, both from WGD and SSD. However, in contrast with “backup without redundancy”, our further examination suggests that functional overlap/redundancy between paralogs is a key determinant of backup capacity between duplicates, with which the buffering potential of any given pair can be accurately predicted. More interestingly, although mutual compensation among duplicate genes is prevalent, we found that the evolution of genetic robustness by gene duplication follows a neutral mode, i.e. the loss of backup capacity being proportional to background mutations accumulated in the divergence time since duplication. Under the neutral mode, although massive duplicates had lost their mutual compensation, we also found natural selection plays a role in maintaining long-term retention of the backup capacity between a few duplicates, which requires slowly evolved functions between paralogs.

## Results

We compiled unambiguous 495 WGD and 667 SSD duplicate pairs from Guan et al. [16], where an improved algorithm on the basis of Kellis et al. [17], [18] was employed to detect WGD paralogs; the independent SSD paralogs were derived from the best reciprocal matches. We removed ribosomal-related duplicates due to their high level of conservation in sequence and expression [19]. In the end, we retained 494 pairs with quantitative genetic interactions from Costanzo et al. [15], which included 266 WGD pairs and 228 SSD pairs (Table S1). The scoring scheme for the synthetic genetic array (SGA) experiment was described in the original publication [15]. Briefly, duplicate pairs showing severer aggravating genetic interactions received more negative interaction scores and were thus deemed to have stronger backup capacity [15]. We note that it is possible that mutual compenstation between some pairs is too subtle to be detected in the current SGA assay, so in this study we only cconsidered pairs with unambiguous genetic interactions revealed by the scoring scheme developed by Costanzo et al. [15].

### Prevalent and strong genetic backup between duplicate paralogs

**Figure 1:**
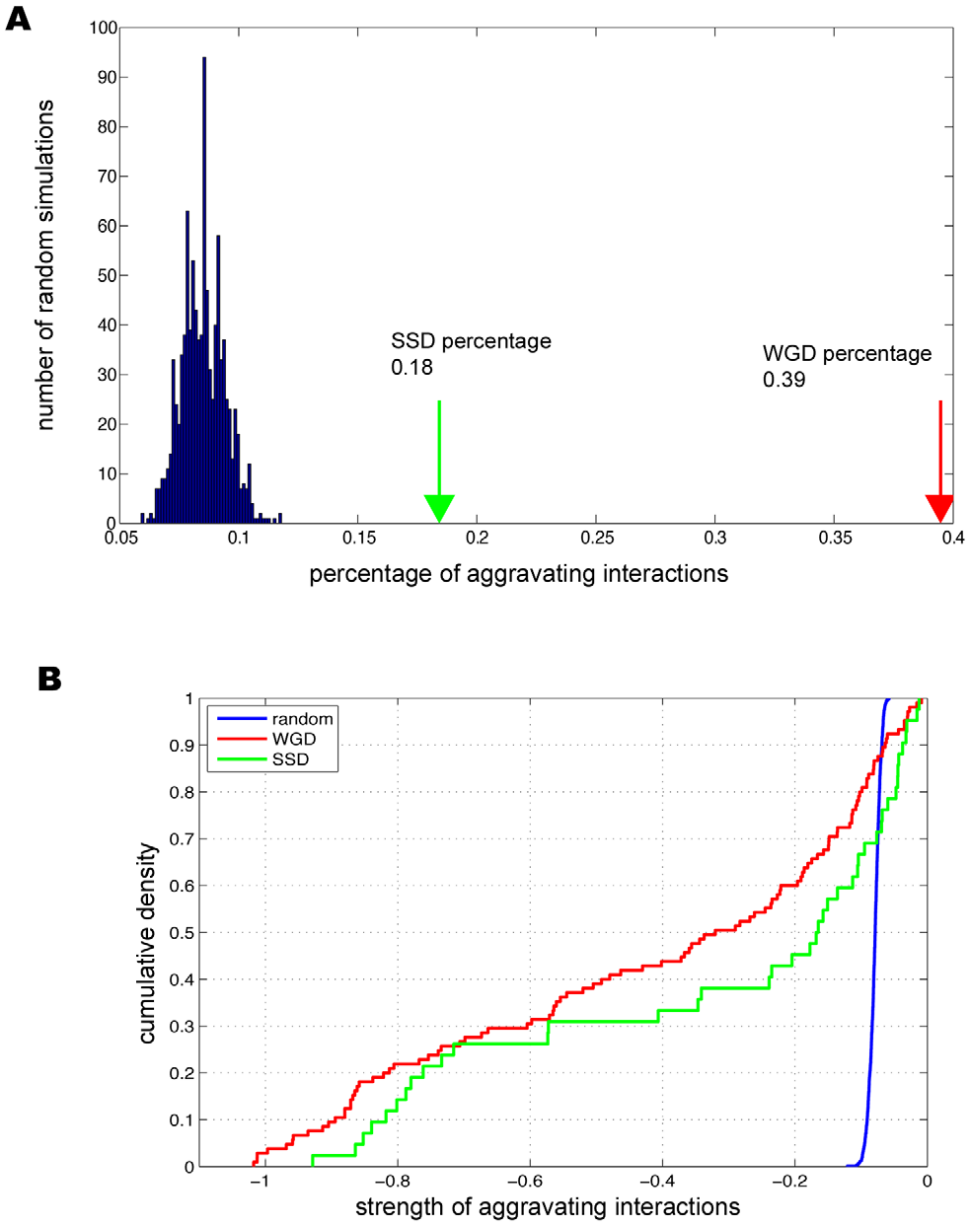
Genetic buffering among duplicated genes. (A) The prevalent genetic buffering between duplicate genes in comparison with the randomly paired genes. (B) Buffering strength between duplicates is stronger than randomly paired genes.

Among the duplicate pairs assayed, we found 39.5% (105/266) of the WGD paralogs had significant aggravating interactions, in comparison with 18.4% (42/228) for SSD paralogs (Figure 1). The percentage of backup pairs for WGD was comparable to what was previously reported (∼35%) [8], where random spore analysis (RSA) and growth curve analysis (GCA) rather than SGA were used to determine the compensatory effects between WGD paralogs. It was interesting that the percentage of backup pairs was much lower for SSD pairs than WGD pairs (Figure 1); such a reduced dispensability of SSD duplicates was previously speculated from single-gene deletion experiments [20].

We further designed two control sets to determine the statistical significance of the observed compensation between duplicated genes. First we randomly chose gene pairs that have genetic interactions regardless of being duplicate or singleton, and found only 7% of the pairs have aggravating interactions (see Figure 1A and Materials and Methods). Second, we took all the duplicated genes and randomly grouped them into pairs, and found that only 6.6% of these random pairs have aggravating interactions. This ruled out the possibility that the observed preferential buffering between duplicates was simply due to that duplicate genes might have more aggravating genetic interactions than singleton genes. Comparing the percentages for the control sets with the percentages of 39.5% for WGD paralogs and 18.4% for SSD paralogs (Figure 1B), our analysis established that duplicates indeed have excessive backup capacity, which results from their intrinsically shared properties. As SGA provides quantitative measurements for the interaction strength between any gene pairs [15], we next studied the backup strength between paralogs. Compared with two control sets, we found the interaction strength between duplicate pairs was much stronger with the average scores of −0.42 and −0.33 for WGD and SSD, respectively, in sharp contrast with −0.07 and −0.069 for the two random control sets, respectively (see Figure 1B, P = 8.54×10^−36^ for WGD, P = 1.87×10^−6^ for SSD and P = 0.06 between WGD and SSD). We note that these findings are in agreement with what was previous reported from analysis on much smaller datasets [6]–[8], [14]. Taken together, our analysis established that strong genetic buffering capacity is prevalent between WGD and SSD paralogs, which provides enhanced genetic robustness in yeast cell.

### Functional similarity is a key determinant of backup capacity between paralogs

Intuitively, genetic robustness by redundancy between gene duplicates should be attributed to their functional similarities. However, conflicting observations were reported in the recent literature [6]–[8], [14]. It was suggested that functional redundancy between buffering duplicates is minimal [8], [14], which gave rise to the hypothesis of “backup without redudancy” [14]. In these earlier studies, functional similarity between paralogs was characterized based on their resemblance in gene expression profiles, protein interactions, or genetic interaction profiles. However, two genes may still buffer each other even though they only have limited functional overlap, which does not require them to have near identical profiles of gene expression or genetic interactions. Supporting this notion, Kafri et al. proposed a model of transcriptional reprogramming, which predicted that differentially expressed duplicates were more likely to buffer each other [21]. Therefore complementary to indirect metrics that score overall functional similarity between duplicate copies (inferred from sequences or expression profiles), a new metric is required to specifically and directly quantify the extent of *functional overlap* between paralogs. In this study, we used a metric called *GO-div* to gauge functional overlap between paralogs directly from their respective GO annotations (see Materials and Methods and also Figure S1). *GO-div* previously was used to benchmark data obtained from high-throughput experiments [22], and here we adopted this approach to quantify functional overlap between duplicate genes. Conceptually, *GO-div* measures the semantic dissimilarity between the sets of Gene Ontology annotations associated with a pair of genes [22] and is calculated on the basis of resemblance between the “best matched” GO terms between sister paralogs, most notably not affected by other diverged functions (see Materials and Methods and also Figure S1 for a schematic illustration). Higher *GO-div* indicates less functional overlap between paralogs while lower *GO-div* indicates both paralogs at least share some very specific functions even though they have diverged in other functions. Although current gene annotations might be incomplete, given the extensive effort in characterizing yeast genes in the past several decades, *GO-div* calibrates *functional overlap* between two genes at least within the best of our current knowledge. Complementary to *GO-div*, we also calculated the non-synonymous substitution rate per site (Ka) between paralogs to represent overall divergence in protein coding sequence between paralogs [23]. Worthy of note, *GO-div* was moderately correlated with Ka with R = 0.2 and P<0.05. The statistical significance indicated their intrinsic consistency in characterizing functional similarity between gene pairs, while the weak correlation suggested that only 4% (R^2^) of the variation in *GO-div* could be explained by Ka, highlighting the non-redundancy of using the two metrics in studying functional divergence.

**Figure 2:**
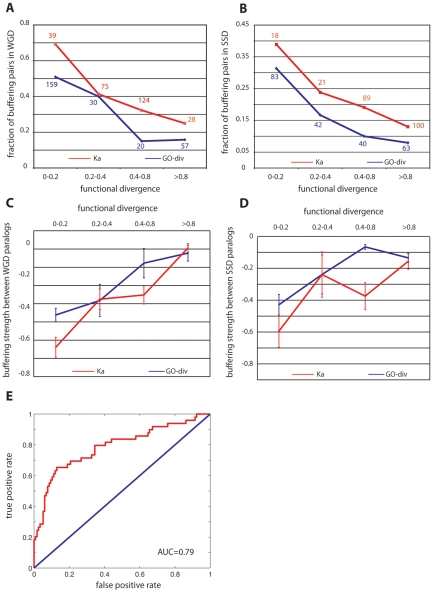
Genetic buffering between gene duplicates results from functional redundancy. (A) and (B) indicate functionally similar genes are more likely to backup each other for WGD (A) and SSD (B) paralogs, respectively, where functional divergence was calibrated by the overlap of GO annotations (*GO-div*) and coding sequence divergence (Ka). The number of total pairs in each bin is indicated for *GO-div* and Ka. (C) and (D) indicate buffering strength between paralogs is on average proportional to their functional similarity for WGD (C) and SSD (D) paralogs, respectively. (E) is the receiver operating characteristic (ROC) curve for the prediction of backup capacity between paralogs based on functional similarities. This curve, together with the AUC (area under the curve) score, was from one random realization of the 3-fold cross-validation.

Among all the duplicate pairs we examined, we found that substantial functional redundancy between paralogs (for both WGD and SSD duplicates) was a key determinant of their genetic backup capability. First, as revealed by Figure 2, duplicate pairs, either WGD (Figure 2A) or SSD (Figure 2B), are more likely to buffer each other if they have less diverged functions; this trend stands when functional divergence was estimated either by the direct measure (*GO-div*) or by ka. Secondly, for the buffering pairs from both WGD and SSD, we found the buffering strength between the paralogs was significantly correlated with their functional divergence (see Figure 2C and 2D) scored by *GO-div*, having Pearson's R = 0.34, P = 3.1×10^−4^ for WGD pairs and R = 0.37, P = 0.01 for SSD pairs. The correlation is also significant when using Ka to approximate functional divergence between paralogs in both WGD and SSD, with Pearson's R = 0.41, P = 1.5×10^−5^ for WGD pairs and R = 0.33, P = 0.03 for SSD pairs. In addition, we also found expression divergence between duplicates (see Materials and Methods) is significantly correlated with their buffering strength for SSD paralogs with R = 0.33, P = 0.03, but not for WGD pairs. This lessened significance of the correlation highlights the superiority of using a direct metric to quantify functional redundancies between duplicates. Taken together, such a tight coupling (see Figure 2C and 2D) between buffering strength and functional overlap between paralogs suggested that the observed prevalent mutual compensation between paralogs (Figure 1) is indeed maintained by their functional similarity, and the less diverged pairs tend to have stronger buffering strength. It is also important to note that WGD and SSD paralogs have different origins and functional propensities [16], [24], therefore our consistent observation on these two classes of duplicates suggested our conclusion was not biased towards particular function categories (as shown in Figure 1 and Figure 2).

Having established the role of genetic redundancy in cellular robustness, we ask whether backup capacity can be predicted for any unseen duplicate pairs. To test this, we pooled the WGD and SSD duplicates together, labeled the 147 pairs (WGD+SSD) that have backup capacity as *positive samples* and the remaining non-backup pairs as *negative*. We characterized each pair with a feature vector, each element being a direct or indirect metric measuring their functional divergence, including Ka, sequence identity, expression divergence and *GO-div*. A support vector machine (SVM) was subsequently implemented to classify these paralogs into either with backup capacity or without. A 3-fold cross-validation, as demonstrated in Figure 2E, suggested that the degree of functional overlap between any paralog pairs was sufficient to distinguish backup pairs from non-backup pairs, with AUC = 0.74±0.05. Such a high predictive power further strengthened our argument that backup between paralogs stems from their functional redundancy. It is also important to note that *GO-div*, which scores the specificity of the best-matched functions between paralogs, is the strongest indicator among all the features to predict backup capacity, and using *GO-div* alone can achieve AUC = 0.7, higher than using combination of any other features (AUC = 0.67).

### Neutral evolution of genetic robustness from gene duplication

**Figure 3:**
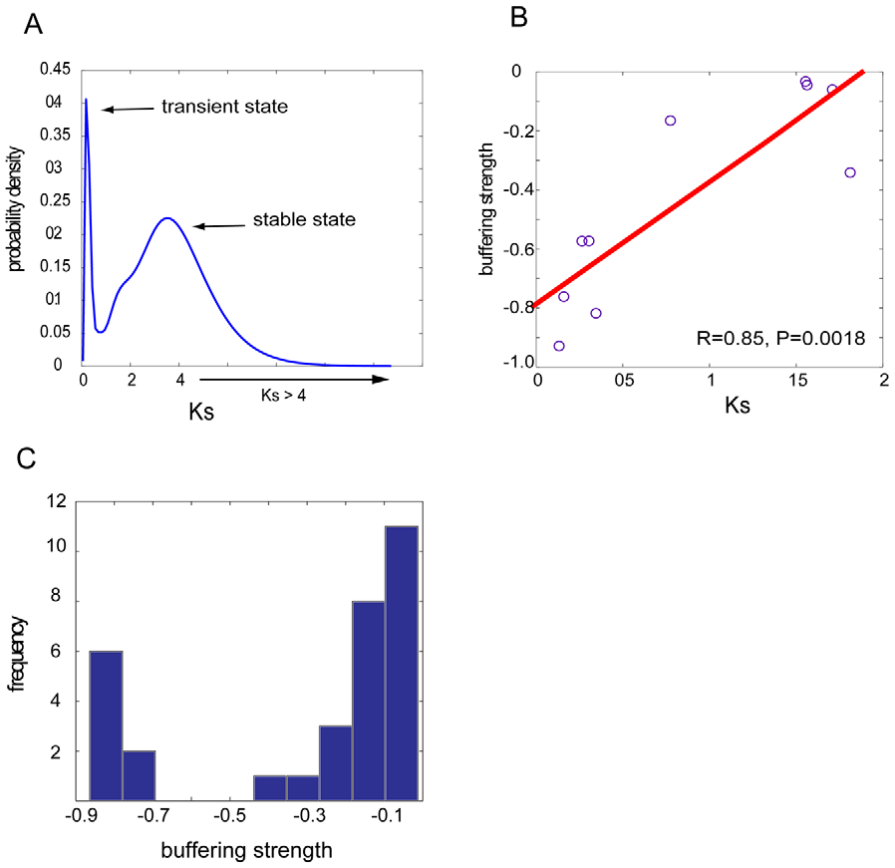
Neutral evolution of genetic buffering between duplicate genes. (A) The distribution of Ks for the SSD buffering pairs shows a bi-modal structure, with one peak on the left representing young duplicates with transient buffering and the other broad peak on the right representing ancient duplicates with stabilized backup by natural selection. The distribution was learned based on kernel density estimation with a Gaussian window. (B) For SSD buffering pairs with Ks<2, their buffering strength is scaled with divergence time, approximated by Ks. (C) Histogram of buffering strength between SSD pairs with Ks>2, indicating ancient pairs could still maintain strong mutual compensation.

In the above we described the presence of prevalent mutual compensation between paralogs (Figure 1) and established that such compensation is maintained by functional overlap (Figure 2). However, such functional redundancy should be understood in a dynamic and evolutionary context because functional similarity between duplicate pairs might be due to a lack of sufficient divergence time, or due to the long-term retention by natural selection. We next decided to delineate the evolutionary trajectory of genetic robustness resulting from gene duplication events. For this purpose, we only considered SSD pairs because they have continuously tractable divergence times, which provide us a dynamic view of genetic robustness in the course of evolution. WGD pairs, however, have all resulted from a single ancient genome duplication event ∼100 millions years ago [17], [25], and thus the observed backup capacity between WGDs have presumably been retained by selection. In the above analysis, we have identified 42 pairs with backup capacity among a total of 228 SSD pairs (Figure 1). We then calculated Ks (the synonymous substitution rate per site in coding sequences) between these buffering SSD paralogs to approximate their divergence time [10], [23]. Strikingly, as revealed in Figure 3A, we found Ks values among these buffering pairs showed a bi-modal distribution. The broad peak on the right (with Ks>2, Figure 3A) represents very ancient paralogs that still maintain their backup capacity. It is known that most paralogs have to functionally diverge to achieve long-term retention [11]; the maintained backup capacity between these ancient pairs should result from severe purifying selection stabilizing their functional redundancy (note that the ribosomal proteins have been removed). The peak on the left, centered at Ks = 0.18, represents very recent duplicates. These recent duplicates have not had sufficient time to functionally diverge, and these very recent paralogs among the buffering pairs may be merely due to an “evolutionary inertia”. In other words, these paralogs are in an evolutionary “transient state” since it is uncertain whether the paralogs will be eventually retained in the genome or whether they could still keep sufficient functional overlap in the course of evolution to maintain mutual backup capacity.

We also examined the remaining 186 SSD pairs, whose mutual compensation had been completely lost; we found the vast majority (88%) is ancient pairs with Ks>2, confirming that the loss of backup capacity needs sufficient divergence time. However, 8 pairs among them showed unusually low Ks values (Ks<1), where 6 pairs are uncharacterized open reading frames or hypothetical proteins with unknown functions. Such a discrepancy might have resulted from rapid loss of functional overlap between these hypothetical proteins. Furthermore, the observation that the majority of the duplicate pairs (186 non-buffering pairs, in comparison with 42 buffering pairs) had lost backup capacity also suggested that maintaining long-term mutual compensation between duplicates is evolutionarily difficult because most mutations affecting fitness are deleterious, and genetic redundancy would be eventually eroded by rampant mutations. Therefore in a neutral mode, it is expected that the loss of buffering strength between paralog pairs should be proportional to the amount of background mutations, scaled by divergence time. However, in the alternative model, which assumes the presence of natural selection, no correlation was expected between these two variables. By using Ks to approximate the amount of background mutations during the divergence time since duplication, we were able to consider 10 pairs with Ks≤2 among the 42 SSD pairs with backup capacity. We did not include gene pairs with Ks greater than 2 since the substitutions might have been saturated, which made it inaccurate to estimate the synonymous rates of substitutions. Interestingly, we found a tight correlation between Ks and the buffering strength between paralogs, with Pearson's correlation R = 0.85, P = 1.8×10^−3^ (Figure 3B), suggesting ∼72% (R^2^) of the variation in backup strength between these duplicates could be explained by Ks. Furthermore, the proportionality of the two variables is characterized by the slope (*k* = 0.41) of the regression line in Figure 3B, suggesting that a 0.41-fold decrease in buffering strength is accompanied with an increase of Ks by every unit. As Ks is highly correlated with Ka, which is an indicator of functional divergence in protein coding sequences, we sought to determine whether Ka was a confounding factor for the correlation between Ks and the buffering strength between the paralogs. We performed a partial correlation analysis; by controlling for the third variable Ka, we found the significant correlation between Ks and the buffering strength still remains (R = 0.64, P = 0.06), while by controlling for Ks, Ka is no longer significantly correlated with the buffering strength (R = 0.41, P = 0.27). This trend is also confirmed on another set of duplicate pairs with no restriction of best reciprocal BLAST matches to include more samples; again significant correlation between Ks and the buffering strength still stands when controlling for Ka (R = 0.68, P = 0.02), while the correlation between Ka and the buffering strength is absent when controlling for Ks (R = 0.32, P = 0.33). This analysis suggested that for duplicate pairs, their gradual loss of mutual buffering strength is scaled by the amount of background mutations (approximated by Ks), not by the non-synonymous mutations (Ka).

To further support this neutral mode of evolution, the buffering WGD pairs serve as a negative control as backup capacity between WGD paralogs has been long-term stabilized by natural selection. In this scenario, the background mutations are expected not to correlate with the buffering strength between duplicate pairs. By considering 18 WGD pairs with mutual compensation and Ks≤2, our partial correlation analysis (after controlling for Ka) consistently confirmed this prediction with R = 0.4 and P = 0.1. Therefore, we concluded that, unless severe natural selection stabilizes genetic redundancy between paralogs for their backup capacity, mutual buffering is generally unstable between paralogs and will be eventually lost given sufficient amount of background mutations, which is proportional to divergence time between paralogs.

For those ancient pairs (Ks>2) that have still retained mutual buffering (the right peak in Figure 3A), some of them exhibit very strong buffering capacity with buffering strength less than −0.7 (Figure 3C). This highlighted the effects of selective pressure in stabilizing functional redundancy between these SSD paralogs. However, it is known that duplicate genes generally have to functionally diverge to achieve long-term retention in the genome [10]–[12]; therefore cells must have adopted some strategies to satisfy these conflicting requirements. One interesting example is an ancient pair *STV1* and *VPH1* with Ks>4. Their coding sequences have significantly diverged (Ka>0.4) but have maintained significant functional overlap with *GO-div* being much smaller than 0.01, manifested by their common function in vacuolar acidification. The protein products of both genes (Stv1p and Vph1p) have a vacuolar-ATPase V0 domain for proton transportation across membranes; however, Stv1p is localized in Golgi and endosomes while Vph1p is localized in vacuole [26]–[29]. Therefore the observed backup capacity between these two paralogs in our study suggests that their function in normal conditions is likely to be specialized for different cellular compartments, but upon perturbations, they could be alternately used to buffer the loss of their respective paralogs. Supporting this scenario, previous experiments have shown that the moderate growth defects of Δ*vph1* mutant could be rescued by over-expression of Stv1p, which led to re-localization of some Stv1p to the vacuole where Vph1p is specifically localized [29]. Therefore this example represents a strategy allowing long-term retention of duplicates by diversifying their sub-cellular localization to retain the same functions, with which functional redundancy between duplicates could be maintained for their long-term mutual buffering.

### Properties of duplicate pairs with retained long-term backup capacity

Lastly, we probed the general genetic properties of these ancient pairs that have maintained their long-term backup capacity. For this purpose we only considered the stabilized buffering paralogs and excluded those transient buffering pairs (such as gene pairs around the left peak in Figure 3A). For WGD pairs, as their mutual compensation remains strong (Figure 1B) even after ∼100 million years of evolution [25], mutual compensation between WGDs is most likely to have been stabilized by natural selection. Therefore we compared the 105 WGD duplicates with retained backup capacity against 161 WGD pairs that had lost their backup capacity. Similarly, for SSD paralogs, we only considered those paralog pairs with sufficient divergence time (Ks>2) and excluded the “transient” buffering paralog pairs since their backup capacity might be eventually lost (see Figure 2A and 2B). In the end, we were able to compare 32 ancient SSD backup pairs (Ks>2) with the 163 non-backup pairs within the same age range (Ks>2).

**Figure 4:**
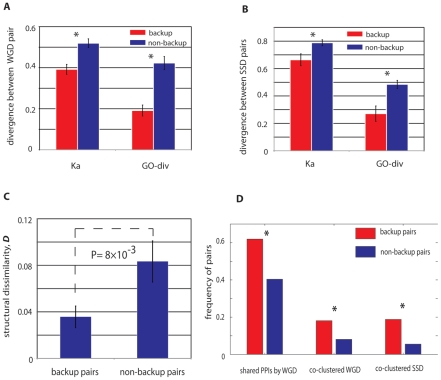
Genetic properties of paralogs with stable mutual buffering. WGD (A) and SSD (B) buffering paralogs have reduced sequence divergence and have more specific overlapping GO annotations. Note that for WGD buffering pairs have more conserved sequence evolution that SSD buffering pairs. (C) The WGD buffering pairs have more conserved structural conformation than the non-buffering pairs. (D) WGD buffering pairs are more likely to share ancestral interacting proteins; both WGD and SSD buffering pairs are more likely to be co-clustered in the same protein complexes.

Compared with the non-buffering paralog pairs, the stabilized buffing paralogs have significantly overlapping functions for both WGD and SSD pairs (20–30% lower than non-backup pairs, Figure 4A and 4B), characterized by *GO-div* and Ka. Particularly for WGD, as they originated from a single duplication event ∼100 million years ago, the observed elevated functional redundancy between the buffering pairs indicates decelerated functional evolution between these duplicates. As the divergence in protein sequence (nonsynonymous substitutions) can also cause divergence in three-dimensional structures, we next examined the difference in secondary structures between these pairs. As expected, we confirmed that functional similarity between buffering pairs from WGD could be also reflected by their structural similarities, with backup pairs usually having similar secondary structural conformations (Figure 4C).

We also collected protein interactions from BioGrid (see Materials and Methods) [30] and found 62% of the backup WGD paralogs have at least one shared interacting protein while the percentage substantially decrease to 40% for non-backup WGD paralogs (Figure 4D, P = 5.95×10^−4^, chi-square test). We performed a similar analysis on the 32 SSD paralogs pairs, but did not find the excessive shared protein interactions in comparison with the matched control. It is likely due to insufficient sample size for SSD backup pairs. In addition, unlike WGD pairs, buffering between SSD pairs is typically weaker than WGD pairs (Figure 1B). Therefore it is likely that the subtle buffering between SSD pairs might not be captured in our analysis of protein interactions. However, regardless of WGD and SSD, we did find the buffering pairs shared some common characteristics. With a total of 392 literature-curated protein complexes examined, we found both WGD and SSD buffering pairs were more likely to be co-clustered in the same protein complexes, with the percentage of ∼18% for the buffering pairs, compared with only ∼5–8% for the non-buffering pairs (see Figure 4D). Worthy of note, previous work showed preferential co-clustering of WGD pairs in protein complexes [31]; thus the further elevated propensity of co-clustering for these buffering WGD pairs reveals a strategy of genetic buffering between duplicates: within the same complex, the backup subunit is always ready to take place of the malfunctioned ones. However, it is important to note that even in the same protein complex, the paralogs still have substantial divergence in sequences and expression profiles, which might indicate the underlying regulatory reprogramming to regulate such a backup strategy [21]. This notion can be best illustrated by one example of a buffering pair derived from SSD, Hos2p (YGL194C) and Rpd3p (YNL330C): both proteins are involved in the histone deacetylase complex; however, Rpd3p is also a member of Rpd3L complex, Rpd3S complex, Sin3 complex and HDB complex. Therefore by differentiating their functions, the two paralogs could achieve long-term retention while their co-clustering in histone deacetylase complex enables their mutual buffering capacity.

## Discussion

There are long-standing debates about the extent and mechanism of genetic robustness contributed by gene duplication. On one hand duplicated genes do show markedly elevated dispensability than singleton genes, which was speculated to result from mutual compensation between paralogs [5]. Alternatively, it was also proposed that such elevated dispensability of duplicates merely results from higher “duplicability” of less important ancestral genes [32]. Therefore, to determine the extent to which yeast paralogs could buffer each other, a systematic interrogation of double-knockouts of yeast paralogs is essential. In this work, we analyzed mutual buffering between yeast paralogs for ∼500 non-redundant WGD and SSD duplicate pairs, a set much larger than what was previously examined. With this largest dataset to this date, we established that merely relying on functional overlap, we are able to accurately predict buffering capacity between paralogs (with AUC>0.74). We further considered the functional redundancy in an evolutionary context, and found recent pairs usually maintain transient functional overlap, and the resulting mutual compensation should be mainly attributed to a lack of sufficient divergence time. However, we also uncovered an appreciable portion of duplicates with long-term retained backup capacity stabilized by selection, which is explained by their conserved functional overlap.

Although both WGD and SSD paralogs could have buffering capacity, substantial difference existed between these two sets, As shown in Figure 1, it is clear that WGD pairs are far more likely to buffer each than SSD pairs (39% vs 18%); WGD pairs also have stronger buffering strength. We reasoned that this disparity might have resulted from differential evolutionary mode between WGD and SSD paralogs [24]. It is known that dosage balance plays an important role in WGD retention [24], [33]; thus the retained WGD paralogs we observed here are expected to be under stronger functional constraints, which reduce the rate of functional divergence between WGD paralogs. Given these facts, their preferential mutual buffering is then anticipated.

While the stoichiometric constraints on WGD pairs provide an explanation to the long-term retained redundancy for backup capacity, we for the first time presented convincing evidence that natural selection also acted on SSD pairs, which were presumably under less stoichiometric constraints compared with WGDs [16], [24]. This finding is interesting as it revealed a different mode of evolution from that of WGD pairs. Without severe stoichiometric constraints, the duplicated copies could have the freedom to experience functional dispersal for their long-term retention, which may bring substantial genetic novelty into an existing system [13]. On the other hand, however, functional overlap between the duplicated copies and the progenitor copies was also selected and therefore stabilized during the course of evolution, which promotes cellular robustness. Though both sides are beneficial for a cell, they are conflicting in nature because most duplicates have to experience substantial functional divergence to achieve their long-term retention [10], [11], and genetic redundancy would be eventually eroded by rampant mutations in the process of functional divergence. However, in our study the observed natural selection on backup capacity between some ancient SSD pairs (Figure 3A and 3C) suggests that in some circumstances, genetic redundancy can still be long-term retained by natural selection, and that cells must have evolved effective strategies to balance the conflicting needs, promoting genetic novelty and systems robustness simultaneously. This point is best demonstrated by the example of *STV1* and *VPH1*, which retained their long-term backup capacity by specializing their overlapping function in different cellular organelles.

Based the results described in this paper, we propose that *genetic redundancy* essentially comes from *functional overlap* between paralogs [7]. This notion is consistently supported in our study by employing *GO-div* to specifically quantify the degree of *functional overlap* between paralogs; this approach is not affected by other differentiated functions that are not shared by the paralogs. In contrast, as shown in previous work, when using expression profiles or genetic interaction profiles to estimate functional divergence on a global scale (in comparison with localized function overlap), strong association between functional similarity and backup capacity is not always observed [14], [31].

Despite the prevalence of mutual compensation between paralogs, our analysis revealed that a large number of duplicate pairs had lost their backup capacity. Indeed, we showed that the erosion of backup capacity between paralogs is essentially a neutral process, with the buffering strength correlated with the amount of background mutations, and proportional to divergence time. Consequently unless evolutionarily stabilized, mutual compensation between most paralogs is an evolutionarily transient state, and cannot substantially contribute to the cellular robustness on a large evolutionary scale. Therefore, beyond genetic redundancy between duplicates, future research is needed to explore other mechanisms contributing to the global robustness in a cell.

## Materials and Methods

### Compiling duplicate genes in budding yeast

We compiled yeast duplicates from Guan et al. [16], where the authors used an improved algorithm to detect paralogs based on Kellis et al [17], [18]. In our study, we studied 495 WGD and 667 SSD paralogs with sequence identity ≥20% as they represent the most confidently assigned paralogs. The SSD pairs were derived from the best reciprocal matches, with one gene being involved in only one pair. Among the WGD and SSD paralogs, we removed the pairs with at least one copy annotated to be ribosomal proteins, and the annotation was based on gene ontology (GO, as of Jan 2009).

### Genetic interaction between paralogs

We mapped the paralogs onto the newly released yeast genetic interaction data generated by high-density synthetic genetic arrays (SGA) [15], and retained a total of 328 pairs that have quantitative genetic interactions. We further complemented this list by quantitating additional 166 pairs with the same platform as Costanzo et al. [15]. In total, we studied 494 non-ribosomal paralogs in this study, in which 266 were WGD paralogs and 228 were SSD paralogs. The scoring scheme for genetic interactions is detailed in Costanzo et al. [15]; the significant negative genetic interactions (interaction score is smaller than 0 and P-val is less than 0.05) between paralogs indicate their mutual backup capacity, implicating that double deletion of a pair induces much sicker growth defect that expected from single-deletions. Therefore the more negative the scores are, the stronger backup capacity is expected.

### Randomization protocols

To determine whether the duplicate pairs have an excess of backup capacity, we generated an ensemble of 1,000 randomized controls. For each control group, we randomly sampled 1,000 gene pairs hat have genetic interaction assayed in Costanzo et al. Then the percentage of random pairs having negative genetic interaction can be determined for each control group, and the distribution of the percentages can be estimated from the 1, 000 randomized controls. To determine whether buffering between paralogs was stronger than random pairs, for each control, we calculated average scores for the pairs maintaining negative interactions, and the distribution of the average scores can be estimated from the 1, 000 randomized controls.

### Calculating sequence divergence

Sequence divergence between paralogs is estimated by synonymous (Ks) and non-synonymous (Ka) substitutions per site by aligning the coding sequences of two genes. We downloaded yeast gene sequences from SGD (Saccharomyces Genome Database), and implemented PAML to calculate Ks and Ka [34].

### Calculating GO–div

Functional similarities between two genes can be measured by their semantic similarities in the Gene Ontology (GO) hierarchy [22], [35]; this approach had been successfully used to benchmark data from high-throughput experiments [36]. In this study, we adopted this approach to quantify functional overlap between duplicate genes. We considered all the GO terms in the hierarchy of Biological Process (BP), and these terms represent a corpus, with which each gene is annotated. We did not consider terms in the hierarchies of Cellular Component (CC) and Molecular Function (MF) because CC is not a direct indicator of functional similarity. MF depicts gene activities at the molecular level, and one or more assemblies of MF define a BP term [37]. Therefore considering BP terms implicitly covers MF annotations. In addition, recent work showed using BP reaches the best performance than using CC and MF terms [22]. Considering annotation quality, we excluded all the electronic annotations (with the code of IEA).

For a duplicate pair, as shown in Figure S1A, copy A is annotated with *m* terms and copy B is annotated with *n* terms, so *GO-div* between copy A and B is defined by:
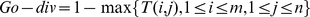
(1)where *T(i,j)* is the semantic similarity between term *i* and *j*.

Calculation of the term-term semantic similarity *T* in a GO hierarchy is demonstrated in Figure S1B by following the protocol described in [22] and [36]. The rationale is that two terms are more similar if they share a very specific ancestral term, and the specificity of a term *x* is defined by the probability, *p(x)*, of randomly sampling the term *x* and all its (recursive) children terms from the BP term collection [35]. With this, the term-term similarity *T(m,n)*, using the term Am for gene copy A and the term Bn for gene copy B as an example (as illustrated in Figure S1A), is defined by:
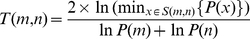
(2)
*S(m,n)* is the set of parent terms shared by *m* and *n* (see Figure S1B), and the numerator of Eq.[2] essentially is to calculate the information content of the most specific parental term(s) shared by *m* and *n* (see Figure S1B). The denominator is a normalization constant to scale the score between 0 and 1. Thus for two terms, if both terms are specific (deep in the GO tree) while their common ancestor term is also very specific, then the two terms receive high score *T*, indicating great semantic similarity between the two terms. At an extreme, when two terms are only overlapped at the root term (Biological Process), then *p(x)* = 1, giving *T* = 0.

Collectively, *GO-div* computes all possible term-term similarity for a duplicate pair (Figure S1A), and scores the best matched GO term pair(s). In this regard, compared with other metrics for characterizing overall functional similarity between duplicates, *GO-div* is more suitable to quantify “*functional overlap*” between paralogs.

### Expression divergence between paralogs

We collected expression profiles for each yeast genes across 549 physiological conditions [38]–[40]. Expression divergence between paralogs is then defined as 1 minus correlation coefficients of expression profiles between sister paralogs.

### SVM implementation

We trained a SVM using RBF kernel with Gaussian variance 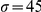 and penalty for soft margin 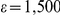.

### Protein secondary structure prediction and comparison

We predicted protein secondary structures using PSIPRED [41], which achieves prediction accuracy >80%. For the predicted structures, we compared the structural resemblance using a newly introduced approach [42], where structural characteristics are encoded in a feature vector comprised of transition probabilities among the basic structural building blocks including α helices, β strands and coils. Information discrepancy between two feature vectors of sister paralogs was calculated to quantify dissimilarity in secondary structures, *D*, and greater *D* indicates more dissimilar secondary structures. This approach has been shown to be robust and objective to classify protein structures [42].

### Compiling protein interaction networks and protein complexes

We downloaded protein-protein interactions from BioGrid (version 2.0.52) [30], and retained protein interactions reported from two-hybrid assays and affinity capture-mass spectrometry. The derived protein interaction network covers 4,873 genes mediate 33, 949 protein interactions. Protein complexes were curated by merging annotations from SGD (Saccharomyces Genome Database), GO (Gene Onotology) and MIPS (The Munich Information Center for Protein Sequences).

## Supporting Information

Figure S1 A schematic illustration of how to calculate *GO-div*. (A) For a duplicate pair with copy A and B, we first get all the annotated terms for each copy, and *GO-div* is calculated on the best matched terms, with the highest term-term similarity. (B) Calculating the term-term similarity in a hierarchical GO tree. For term *m* and *n*, in this example, their semantic similarity is measured by their most specific common ancestor. Term specificity is calibrated by the probability of randomly sampling a term and all its associated children terms from the global GO hierarchy (indicated by *p* in the figure). The term set *S* represents the common ancestral nodes between node *m* and *n*.
(0.39 MB PDF)

Table S1 The duplicate pairs analyzed in this study, grouped by WGD and SSD separately.
(0.29 MB PDF)
