# Supplementary material for: The Cellular Robustness by Genetic Redundancy in Budding Yeast
Source: PLoS Genet. 2010 Nov 4;6(11):e1001187. doi: 10.1371/journal.pgen.1001187 (PMC2973813; doi:10.1371/journal.pgen.1001187)
Supplement: Figure S1 — A schematic illustration of how to calculate GO-div. (A) For a duplicate pair with copy A and B, we first get all the annotated terms for each copy, and GO-div is calculated on the best matched terms, with the highest term-term similarity. (B) Calculating the term-term similarity in a hierarchical GO tree. For term m and n, in this example, their semantic similarity is measured by their most specific common ancestor. Term specificity is calibrated by the probability of randomly sampling a term and all its associated children terms from the global GO hierarchy (indicated by p in the figure). The term set S represents the common ancestral nodes between node m and n. (0.39 MB PDF) [file pgen.1001187.s001.pdf]

A

copy B

copy A

	Term B1	Term B2	···	Term Bn
Term A1	T(1,1)	T(1,2)	···	T(1,n)
Term A2	T(1,2)			
···	···			
Term Am	T(1,m)	T(2,m)		T(m,n)

$$GO-div = 1-\max\{T(i,j), 1\le i\le m, 1\le j\le n\}$$

B

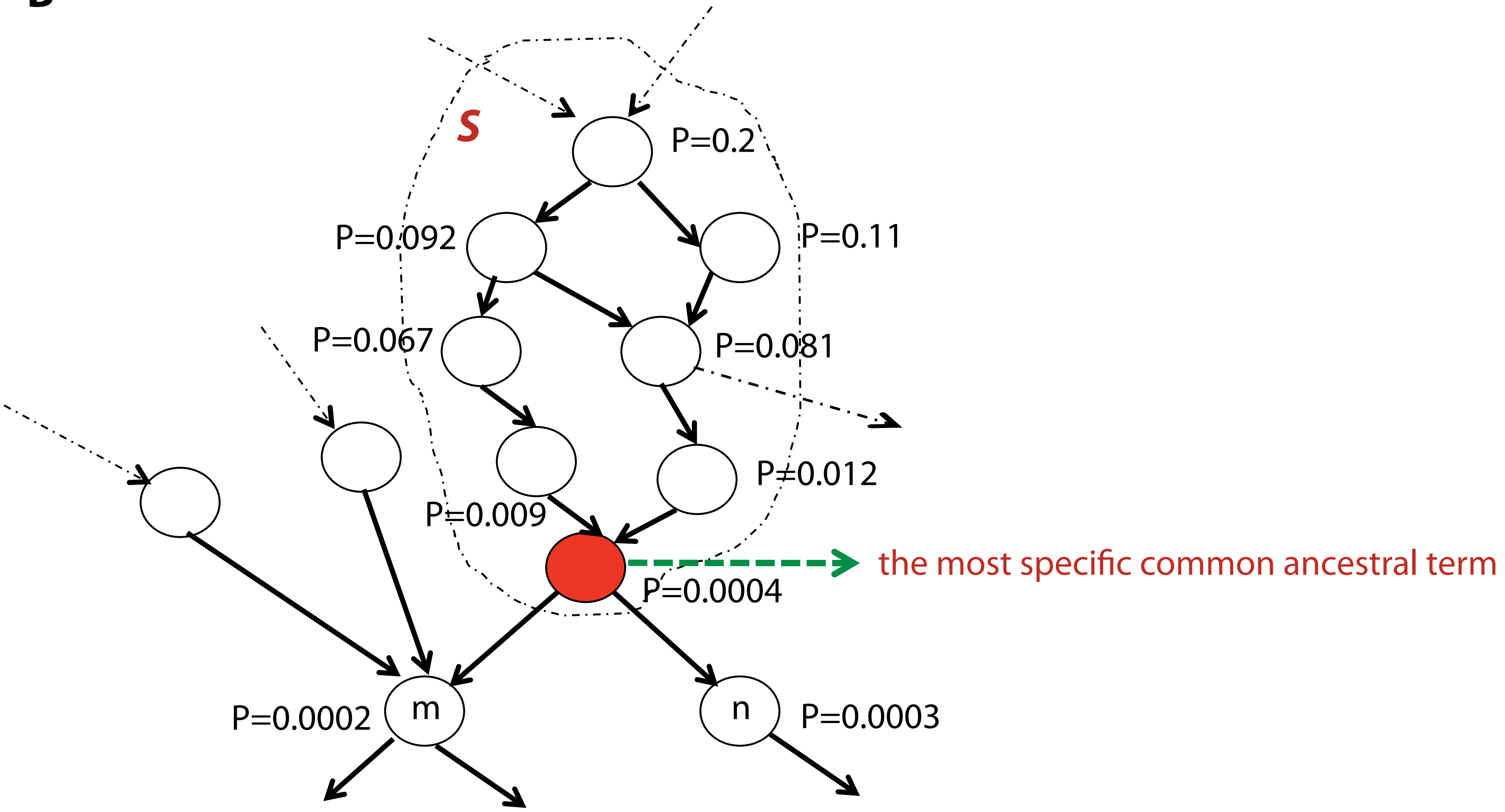

$$T(m,n)=\frac{2\times\ln(\min_{x\in S(m,n)}\{P(x)\})}{\ln P(m)+\ln P(n)}=\frac{2\times\ln(0.0004)}{\ln(0.0002)+\ln(0.0003)}=0.94$$

Fig S1
