# Supplementary material for: The Cellular Robustness by Genetic Redundancy in Budding Yeast
Source: PLoS Genet. 2010 Nov 4;6(11):e1001187. doi: 10.1371/journal.pgen.1001187 (PMC2973813; doi:10.1371/journal.pgen.1001187)
Supplement: Table S1 — The duplicate pairs analyzed in this study, grouped by WGD and SSD separately. (0.29 MB PDF) [file pgen.1001187.s002.pdf]

Table S1 Duplicate pairs analyzed in this study

Score: genetic interaction score for each interaction
P-value: the P-value associated with each interaction
GO-div: functional divergence gauged by GO annotations
Backup: this column indicates whether the two copies have mutual compensation
Pairs are grouped by WGDs and SSDs.

	**Duplicated Gene Pairs**		**Score**	**P-value**	***GO-div***	***Backup***
	**ORF 1**	**ORF2**				
WGD	YIR018W	YOL028C	0.0246	2.76E-02	0.109918	NO
	YKR091W	YOR083W	0.1173	7.22E-02	0.648995	NO
	YLR266C	YOR172W	-0.0263	2.42E-01	0.109918	NO
	YER035W	YGL222C	-0.0635	4.05E-02	0.105015	YES
	YBL059W	YER093C-A	0.074	2.01E-01	1	NO
	YMR198W	YPL253C	-0.1141	2.07E-02	0.124806	YES
	YGR142W	YPR158W	-0.0211	3.10E-01	1	NO
	YBR273C	YJL048C	-0.0016	4.84E-01	0.034069	NO
	YBR182C	YPL089C	-0.0179	3.34E-01	0.109918	NO
	YMR195W	YPL250C	-0.0789	6.15E-02	1	NO
	YGR041W	YLR353W	-0.0065	3.97E-01	0.138342	NO
	YBL054W	YER088C	-0.0014	4.90E-01	0.02175	NO
	YDR206W	YLR233C	-0.0098	3.32E-01	0.026517	NO
	YMR119W	YNL008C	0.0279	3.08E-01	0.034069	NO
	YIL149C	YKR095W	-0.2227	0.00E+00	0.007526	YES
	YGR071C	YLR373C	-0.0268	7.19E-03	1	YES
	YHL034C	YLL046C	-0.0277	2.92E-01	0.937511	NO
	YER001W	YIL014W	0.0212	8.21E-02	0.17108	NO
	YDR492W	YOL101C	0.0094	4.51E-01	0.012954	NO
	YGR188C	YJL013C	0.0233	2.08E-01	0.112086	NO
	YBR150C	YOL089C	0.0428	7.47E-02	1	NO
	YGL133W	YPL216W	0.0244	2.19E-01	1	NO
	YKL062W	YMR037C	-0.0988	3.13E-12	0.019245	YES
	YDR146C	YLR131C	0.0789	9.04E-04	0.0761	NO
	YHR133C	YNL156C	0.0136	2.67E-01	0.100999	NO
	YDL226C	YNL204C	0.0003	4.98E-01	0.935858	NO
	YFL042C	YLR072W	-0.0437	2.94E-02	1	YES
	YDR309C	YHR061C	-0.0887	5.15E-54	0.068613	YES
	YBL009W	YGL021W	-0.0304	3.40E-01	0.018392	NO
	YDL204W	YDR233C	-0.0135	1.49E-01	1	NO
	YDR379W	YOR127W	-0.0258	2.74E-01	0.044869	NO
	YJL105W	YKR029C	-0.0284	1.87E-01	1	NO
	YKL133C	YMR115W	-0.0348	3.95E-02	1	YES
	YML109W	YMR273C	-0.9969	0.00E+00	0.043082	YES
	YDL224C	YNL197C	-0.2213	3.96E-13	0.051486	YES

	YDL088C	YMR153W	-0.5203	6.80E-45	0.022256	YES
	YDR458C	YML034W	0.0195	2.46E-01	1	NO
	YCL051W	YDR528W	0.017	4.25E-01	0.21364	NO
	YDR501W	YLR183C	-0.0296	2.88E-05	0.003666	YES
	YDR423C	YML007W	0.0213	3.35E-01	0.109918	NO
	YGR213C	YLR046C	-0.0158	2.27E-02	1	YES
	YBL101C	YPR030W	-0.0328	1.60E-01	0.21364	NO
	YCR069W	YNR028W	0.0299	1.05E-01	1	NO
	YDR409W	YOR156C	-0.4922	3.98E-47	0.239436	YES
	YBR216C	YGL060W	-0.0108	3.72E-01	1	NO
	YGL071W	YPL202C	-0.6985	5.79E-117	0.084482	YES
	YJL112W	YKR036C	-0.1267	6.01E-02	0.067176	NO
	YBL005W	YGL013C	0.0211	3.72E-01	0.109918	NO
	YGL134W	YPL219W	-0.0142	3.08E-01	0.112086	NO
	YGL162W	YPR009W	-0.0335	3.36E-01	0.02175	NO
	YBR172C	YPL105C	-0.0394	2.54E-01	1	NO
	YDR451C	YML027W	-0.0289	1.08E-01	0.147278	NO
	YDR066C	YER139C	0.0209	2.36E-01	1	NO
	YHR155W	YNL257C	0.0311	8.07E-02	1	NO
	YDR438W	YML018C	0.0075	3.91E-01	1	NO
	YDR147W	YLR133W	-0.0203	3.17E-01	0.033056	NO
	YBL091C-A	YER120W	0.0158	1.57E-05	0.116411	NO
	YGL166W	YPR008W	-0.0081	4.14E-01	0.067176	NO
	YDR251W	YPL032C	0.0328	3.00E-01	0.078975	NO
	YAL056W	YOR371C	-0.191	4.24E-04	0.019245	YES
	YGR023W	YLR332W	0.0808	1.08E-02	0.204796	NO
	YDR463W	YHR006W	-0.4018	0.00E+00	0.084482	YES
	YBL085W	YER114C	-1.0123	0.00E+00	0.0761	YES
	YBL061C	YER096W	-0.1037	0.00E+00	0.156666	YES
	YOR229W	YPL139C	-0.0114	3.10E-01	0.034069	NO
	YBR014C	YDL010W	0.0479	3.08E-01	0.119448	NO
	YER054C	YIL045W	0.0138	2.47E-01	0.046904	NO
	YDR264C	YOR034C	-0.0006	4.84E-01	0.013855	NO
	YFL018C	YPL017C	-0.1139	2.70E-02	0.740324	YES
	YDL214C	YNL183C	0.0008	4.93E-01	0.615093	NO
	YFR022W	YOR018W	0.0822	9.63E-02	0.410687	NO
	YKR075C	YOR062C	0.0404	1.63E-01	1	NO
	YKL043W	YMR016C	-0.245	0.00E+00	0.182804	YES
	YJL165C	YKL168C	-0.0362	2.67E-01	1	NO
	YAL051W	YOR363C	-0.0182	1.85E-01	0.01546	NO
	YDR389W	YOR134W	-0.0073	4.52E-01	0.332265	NO
	YAL015C	YOL043C	0.0103	3.01E-01	0.097358	NO
	YJL110C	YKR034W	-0.0098	4.20E-01	0.033056	NO
	YIR033W	YKL020C	-0.5544	3.64E-20	0.109918	YES
	YKL121W	YMR102C	0.0006	4.94E-01	1	NO
	YGL063W	YPL212C	-0.0747	2.01E-22	0.13754	YES

	YNL307C	YOL128C	0.0282	2.93E-01	0.121446	NO
	YCL036W	YDR514C	0.0112	3.79E-01	1	NO
	YDR132C	YLR108C	-0.0055	4.61E-01	1	NO
	YDR151C	YLR136C	-0.0227	1.48E-01	0.009819	NO
	YDR096W	YER169W	-0.0604	3.73E-04	0.01271	YES
	YJR054W	YML047C	-0.1217	2.79E-01	1	NO
	YER129W	YGL179C	-0.0313	2.93E-01	0.121446	NO
	YBR042C	YDR018C	-0.0041	4.77E-01	0.116411	NO
	YFR023W	YHR015W	0.0127	4.35E-01	0.939109	NO
	YCR026C	YEL016C	0.0342	2.50E-01	0.292042	NO
	YDR351W	YHR103W	-0.1061	1.22E-06	0.21364	YES
	YBR066C	YDR043C	-0.062	8.16E-05	0.182804	YES
	YGR238C	YHR158C	-0.037	3.06E-01	0.198532	NO
	YJR061W	YKL201C	0.0104	4.23E-01	1	NO
	YBR284W	YJL070C	-0.095	1.09E-01	1	NO
	YJL098W	YKR028W	-0.3669	6.11E-68	0.126235	YES
	YDR253C	YPL038W	-0.868	5.35E-107	0.228237	YES
	YCL048W	YDR522C	-0.0165	7.68E-02	0.141437	NO
	YDR200C	YLR238W	0.0491	1.38E-02	0.070718	NO
	YGL082W	YPL191C	0.0078	4.46E-01	1	NO
	YJL099W	YKR027W	-0.1569	4.39E-03	0.057373	YES
	YDL175C	YIL079C	-0.9591	0.00E+00	0.058696	YES
	YIL113W	YNL053W	-0.0039	3.66E-01	0.467884	NO
	YGL158W	YLR248W	-0.0184	3.38E-01	0.034069	NO
	YER132C	YGL197W	-0.4302	3.48E-13	0.178214	YES
	YML118W	YMR285C	-0.0608	1.88E-01	1	NO
	YBR270C	YJL058C	0.0589	1.11E-05	1	NO
	YOR233W	YPL141C	-0.2268	6.17E-08	1	YES
	YBL067C	YER098W	-0.0929	1.12E-01	1	NO
	YGR004W	YLR324W	0.0147	1.92E-01	0.578441	NO
	YGR197C	YJR015W	-0.0134	3.76E-01	1	NO
	YIL156W	YKR098C	-0.0013	4.36E-01	1	NO
	YBR005W	YDR003W	-0.0025	4.72E-01	0.422263	NO
	YCL011C	YNL004W	-0.1361	4.52E-18	0.164203	YES
	YKL072W	YMR053C	-0.0913	9.78E-04	1	YES
	YDL109C	YGL144C	-0.0041	4.60E-01	0.012954	NO
	YDR144C	YLR120C	-0.0415	8.79E-02	0.008221	NO
	YDR348C	YHR097C	-0.0828	6.33E-02	1	NO
	YER037W	YGL224C	-0.0052	4.69E-01	1	NO
	YDR247W	YPL026C	-0.014	3.44E-01	0.121446	NO
	YIL131C	YNL068C	-0.1023	1.46E-13	0.088236	YES
	YDR213W	YLR228C	-0.8946	2.32E-72	0.100999	YES
	YJR059W	YKL198C	-0.3374	7.04E-22	0.463079	YES
	YFR040W	YGL229C	-0.0094	3.99E-01	0.205857	NO
	YHR003C	YKL027W	0.0544	1.82E-01	1	NO
	YCR048W	YNR019W	-0.1491	8.67E-03	0.318845	YES

	YCL024W	YDR507C	-0.0418	2.75E-01	0.048182	NO
	YIL095W	YNL020C	-1.0126	0.00E+00	0.055545	YES
	YDL048C	YLR375W	0.0007	4.75E-01	1	NO
	YGR248W	YHR163W	-0.0348	1.17E-02	0.141437	YES
	YKR072C	YOR054C	-0.6637	7.73E-128	0.0761	YES
	YDL155W	YLR210W	-0.0311	3.36E-01	0.059543	NO
	YDR252W	YPL037C	-0.0411	2.41E-01	0.164203	NO
	YDR178W	YLR164W	-0.0195	3.04E-01	1	NO
	YGR109C	YPR120C	-0.1961	0.00E+00	0.058696	YES
	YHL017W	YKL039W	0.0071	3.28E-01	1	NO
	YDL222C	YNL194C	-0.1886	1.94E-02	0.092024	YES
	YDR358W	YHR108W	-0.2346	2.43E-45	0.070718	YES
	YGR070W	YLR371W	-0.545	3.35E-25	0.048182	YES
	YJL134W	YKR053C	0.0143	3.83E-01	0.030845	NO
	YER059W	YIL050W	-0.0251	2.47E-01	0.033056	NO
	YDR505C	YLR177W	-0.0202	5.74E-02	1	NO
	YBL089W	YER119C	0.0174	1.93E-01	0.370545	NO
	YHR117W	YNL121C	-0.2613	2.47E-04	1	YES
	YDR263C	YOR033C	0.0043	4.74E-01	0.214399	NO
	YML111W	YMR275C	-0.2901	2.37E-11	0.258156	YES
	YIL085C	YNL029C	0.0058	4.40E-01	0.210541	NO
	YIL105C	YNL047C	-0.0798	1.85E-01	0.042592	NO
	YHR030C	YKL161C	0.0166	4.01E-01	1	NO
	YCR073C	YNR031C	-0.0123	4.18E-01	0.098262	NO
	YBR183W	YPL087W	-0.0155	3.44E-01	0.05895	NO
	YNL298W	YOL113W	-0.0531	1.85E-01	0.068613	NO
	YBR078W	YDR055W	0.0609	1.86E-01	0.21364	NO
	YEL060C	YOR003W	0.0125	3.94E-01	0.316565	NO
	YBR210W	YGL054C	-0.2831	1.95E-07	0.112086	YES
	YHR123W	YNL130C	-0.0915	3.77E-02	0.242341	YES
	YDR185C	YLR168C	-0.166	8.11E-03	0.378793	YES
	YML100W	YMR261C	-0.0938	1.37E-01	0.182804	NO
	YBR052C	YDR032C	0.0153	3.35E-01	1	NO
	YCR034W	YJL196C	-0.0434	2.66E-01	0.257158	NO
	YBL106C	YPR032W	-0.3597	7.22E-04	0.062676	YES
	YIR037W	YKL026C	0.02	3.69E-01	0.54559	NO
	YJL160C	YKL164C	-0.03	3.15E-06	1	YES
	YCR091W	YNR047W	-0.0661	6.28E-02	0.099365	NO
	YDR122W	YLR096W	-0.0674	3.29E-03	0.121446	YES
	YMR243C	YOR316C	-0.4633	2.11E-13	0.044516	YES
	YCR094W	YNR048W	-0.0041	4.08E-01	1	NO
	YBR177C	YPL095C	0.005	3.38E-01	0.112086	NO
	YFR014C	YOL016C	-0.0152	3.44E-01	0.121446	NO
	YAL017W	YOL045W	0.0346	8.07E-03	0.070718	NO
	YGL084C	YPL189W	0.0406	2.37E-01	0.159291	NO
	YJR130C	YML082W	-0.0225	3.52E-01	0.670398	NO

	YDR441C	YML022W	0.0108	3.90E-01	1	NO
	YDL085W	YMR145C	0.05	4.64E-102	0.112086	NO
	YNL093W	YOR089C	-0.1495	2.54E-04	0.014806	YES
	YNL098C	YOR101W	-0.8712	2.31E-82	0.01862	YES
	YGR108W	YPR119W	-0.88	2.17E-69	0.059543	YES
	YBR147W	YOL092W	-0.0179	3.41E-01	1	NO
	YDR436W	YML016C	-0.268	1.48E-05	0.044516	YES
	YDR272W	YOR040W	-0.0089	1.25E-02	0.041073	YES
	YMR199W	YPL256C	-0.108	3.98E-02	0.059543	YES
	YDR277C	YOR047C	-0.0681	9.99E-03	0.131148	YES
	YCL050C	YDR530C	-0.0404	2.62E-01	0.000673	NO
	YOR231W	YPL140C	-0.2364	9.86E-40	0.121446	YES
	YLR450W	YML075C	-0.8216	3.55E-149	0.026517	YES
	YBR214W	YGL056C	-0.0572	2.05E-01	0.24588	NO
	YLR449W	YML074C	-0.0809	4.06E-12	0.706283	YES
	YMR246W	YOR317W	-0.0185	3.54E-01	0.012954	NO
	YMR008C	YOL011W	0.0165	2.70E-01	0.136858	NO
	YDR111C	YLR089C	0.0451	6.57E-02	1	NO
	YOR222W	YPL134C	-0.016	3.59E-01	0.400361	NO
	YJL139C	YKR061W	-0.1358	5.48E-03	0.070718	YES
	YBL056W	YER089C	-0.1128	3.96E-02	0.141437	YES
	YBR016W	YDL012C	0.0291	1.57E-01	1	NO
	YDL079C	YMR139W	0.0507	5.83E-02	0.121446	NO
	YBR242W	YGL101W	-0.0121	3.12E-01	1	NO
	YGL089C	YPL187W	-0.0632	1.14E-01	0.044869	NO
	YFR024C-A	YHR016C	0.0334	5.02E-02	0.055545	NO
	YCL025C	YDR508C	-0.5655	1.84E-24	0.107564	YES
	YHR135C	YNL154C	-0.8127	0.00E+00	0.014806	YES
	YJL116C	YKR042W	-0.0412	1.11E-01	0.435345	NO
	YLL062C	YPL273W	-0.0174	2.31E-01	0.348317	NO
	YGR136W	YPR154W	0.0984	1.84E-01	0.26584	NO
	YLR433C	YML057W	-0.0252	1.35E-01	0.008781	NO
	YAL023C	YOR321W	-1.0178	0.00E+00	0.075012	YES
	YLR300W	YOR190W	-0.0239	2.88E-01	0.932673	NO
	YCL035C	YDR513W	0.0151	1.35E-01	0.514556	NO
	YDR098C	YER174C	-0.8583	0.00E+00	0.022564	YES
	YBR161W	YPL057C	-0.5977	2.39E-28	0.030845	YES
	YJL082W	YKR018C	-0.3719	1.10E-07	1	YES
	YCR037C	YJL198W	0.0108	4.19E-01	0.243627	NO
	YDR368W	YOR120W	0.0039	4.61E-01	0.096531	NO
	YBR104W	YPR058W	-0.03	2.34E-01	0.400361	NO
	YBR054W	YDR033W	0.0328	3.69E-05	1	NO
	YIL123W	YNL066W	-0.0194	2.12E-01	0.634175	NO
	YAL007C	YOR016C	0.0048	4.77E-01	0.234415	NO
	YIL138C	YNL079C	-0.7344	9.19E-45	0.0761	YES
	YGR038W	YLR350W	-0.6046	7.94E-32	0.062873	YES

	YGR043C	YLR354C	-0.032	1.53E-01	0.616133	NO
	YBR117C	YPR074C	-0.1806	1.78E-52	0.048182	YES
	YJL164C	YKL166C	-0.1747	2.11E-03	0.01862	YES
	YJL133W	YKR052C	-0.3204	2.68E-68	0.258583	YES
	YLL061W	YPL274W	0.0072	3.97E-01	0.212155	NO
	YHR047C	YKL157W	-0.019	2.19E-01	0.596005	NO
	YBR068C	YDR046C	-0.025	3.12E-01	0.107564	NO
	YCR073W-A	YNR034W	-0.1862	6.96E-03	0.0761	YES
	YML106W	YMR271C	-0.0314	3.08E-01	0.130551	NO
	YDR107C	YLR083C	-0.0458	3.15E-01	0.182804	NO
	YAL030W	YOR327C	-0.1483	1.11E-03	0.014806	YES
	YGR010W	YLR328W	-0.9684	0.00E+00	0.093623	YES
	YBR020W	YDR009W	0.0121	3.37E-01	0.100599	NO
	YKL129C	YMR109W	-0.9357	3.65E-140	0.014806	YES
	YGR121C	YPR138C	0.011	4.21E-01	0.176699	NO
	YOR226C	YPL135W	-0.8378	0.00E+00	0.022564	YES
	YBR169C	YPL106C	-0.3561	6.40E-34	0.205504	YES
	YGR092W	YPR111W	-0.4798	4.16E-15	0.121446	YES
	YMR183C	YPL232W	-0.7678	5.61E-80	0.062676	YES
	YBR001C	YDR001C	-0.0817	3.82E-15	0.212155	YES
	YGR209C	YLR043C	-0.5604	0.00E+00	0.062754	YES
	YDR497C	YOL103W	-0.5709	0.00E+00	0.159291	YES
	YKL127W	YMR105C	-0.3442	1.18E-10	0.070718	YES
	YBL039C	YJR103W	-0.7538	0.00E+00	0.079646	YES
	YFR053C	YGL253W	-0.7389	1.45E-28	0.070718	YES
	YER031C	YGL210W	-0.8059	2.58E-122	0.214109	YES
	YFR015C	YLR258W	0.0368	2.01E-01	0.075012	NO
	YER070W	YIL066C	0.0331	3.19E-01	0.154725	NO
	YNL104C	YOR108W	0.0144	2.20E-01	0.112086	NO
	YJL045W	YKL148C	-0.0645	8.63E-02	0.141909	NO
	YDR453C	YML028W	0.02	3.02E-03	0.48889	NO
	YGR124W	YPR145W	-0.1173	6.57E-03	0.120211	YES
	YLR432W	YML056C	-0.0804	2.75E-07	0.081395	YES
	YGR032W	YLR342W	-0.5047	9.74E-29	0.068613	YES
	YGR138C	YPR156C	-0.0602	1.72E-01	0.141437	NO
	YDR502C	YLR180W	-0.8801	0.00E+00	0.148084	YES
	YBR218C	YGL062W	-0.5642	2.63E-11	0.063775	YES
	YER062C	YIL053W	-0.9134	0.00E+00	0.138342	YES
	YBR082C	YDR059C	-0.8614	0.00E+00	0.226945	YES
	YDR099W	YER177W	-0.7065	1.83E-110	0.01094	YES
	YCR024C-A	YEL017C-A	-0.0188	1.26E-01	0.108895	NO
	YDR312W	YHR066W	-0.865	0.00E+00	0.044516	YES
	YGR254W	YHR174W	-0.6735	0.00E+00	0.070718	YES
	YGR192C	YJR009C	-0.9045	0.00E+00	0.049889	YES
	YMR186W	YPL240C	-0.8711	5.56E-123	0.116639	YES
	YCL069W	YKR105C	0.0181	2.90E-01	0.579294	NO

	YDL229W	YNL209W	-0.9579	0.00E+00	0.079646	YES
SSD	YEL070W	YNR073C	-0.0111	3.48E-01	1	NO
	YBR010W	YNL031C	-0.5725	0.00E+00	0.024791	YES
	YBR009C	YNL030W	-0.9286	0.00E+00	0.259727	YES
	YAL068C	YGL261C	-0.0004	4.85E-01	1	NO
	YBR299W	YGR292W	-0.0079	3.15E-01	0.112086	NO
	YBR301W	YGR294W	0.0504	3.35E-03	1	NO
	YBL003C	YDR225W	-0.5733	0.00E+00	0.043681	YES
	YAL005C	YLL024C	-0.7616	1.14E-49	0.164203	YES
	YDL243C	YFL057C	-0.0111	1.92E-01	0.525796	NO
	YCR102C	YLR460C	-0.0535	1.76E-01	1	NO
	YAL063C	YAR050W	0.0466	2.89E-01	0.13754	NO
	YLR044C	YLR134W	-0.8179	0.00E+00	0.057373	YES
	YER011W	YOR010C	-0.0111	3.77E-01	0.367768	NO
	YLR237W	YOR192C	-0.1648	2.87E-13	0.247168	YES
	YDR345C	YHR094C	-0.0063	4.13E-01	0.138336	NO
	YHR179W	YPL171C	0.0093	3.46E-01	1	NO
	YFL011W	YMR011W	0.0107	4.20E-01	0.138336	NO
	YJL159W	YKL164C	-0.3414	2.11E-07	0.21364	YES
	YBR244W	YIR037W	-0.0251	2.14E-01	0.54559	NO
	YDL037C	YIR019C	0.0129	2.79E-01	0.951353	NO
	YBR084W	YGR204W	-0.0124	1.68E-13	0.224121	YES
	YDL066W	YLR174W	-0.0445	2.25E-04	0.212155	YES
	YDR155C	YML078W	-0.04	6.11E-02	0.461598	NO
	YGR086C	YPL004C	-0.0083	3.33E-01	0.014806	NO
	YBR294W	YLR092W	0.0598	8.35E-02	0.277298	NO
	YIL169C	YOL155C	-0.0209	2.96E-01	1	NO
	YMR253C	YPL264C	0.0422	4.37E-03	1	NO
	YKL166C	YPL203W	0.0699	1.73E-02	0.01862	NO
	YCR105W	YMR318C	0.0159	2.31E-01	0.702886	NO
	YDR534C	YOR383C	-0.0135	1.98E-01	0.670121	NO
	YER073W	YOR374W	-0.028	3.02E-01	0.201188	NO
	YBR249C	YDR035W	-0.0284	2.57E-01	0.024313	NO
	YBR263W	YLR058C	-0.0581	2.41E-01	0.673223	NO
	YDR541C	YOL151W	-0.0015	4.64E-01	1	NO
	YIL035C	YOR061W	-0.7878	1.39E-71	0.008781	YES
	YDR399W	YJR133W	-0.113	1.02E-47	0.164203	YES
	YKR104W	YLL048C	-0.0085	4.21E-01	0.287342	NO
	YBR298C	YGR289C	0.0242	2.35E-02	0.150948	NO
	YGR032W	YMR306W	-0.0224	3.12E-01	0.141437	NO
	YBR164C	YDL192W	-0.239	3.13E-05	0.06794	YES
	YDR287W	YHR046C	0.0311	3.74E-01	0.044516	NO
	YKL046C	YMR238W	-0.78	2.39E-57	0.048182	YES
	YDL137W	YOR094W	0.0754	8.32E-02	0.117033	NO
	YMR054W	YOR270C	-0.1775	8.86E-08	0.000673	YES
	YDR139C	YLL039C	-0.0432	5.24E-05	0.226945	YES

	YHR050W	YLR034C	-0.0254	2.17E-01	0.231179	NO
	YGR132C	YGR231C	0.2212	2.93E-65	0.035916	NO
	YBR069C	YCL025C	-0.0304	1.69E-01	0.107564	NO
	YLR146C	YPR069C	0.0432	1.51E-01	0.058696	NO
	YDR519W	YNL135C	0.0081	4.48E-01	1	NO
	YBR205W	YOR099W	0.0065	3.95E-01	0.17108	NO
	YDL078C	YKL085W	-0.0028	4.78E-01	0.436794	NO
	YBR245C	YOR304W	-0.0128	4.06E-01	0.01546	NO
	YER123W	YNL154C	-0.0692	1.31E-02	0.121446	YES
	YBR025C	YHL014C	-0.0321	2.53E-01	1	NO
	YFL041W	YMR058W	-0.0066	4.52E-01	0.231179	NO
	YFL053W	YML070W	-0.0047	4.45E-01	0.084612	NO
	YAL023C	YGR199W	-0.8014	0.00E+00	0.17108	YES
	YML120C	YMR145C	-0.0473	2.63E-02	0.112086	YES
	YNR001C	YPR001W	-0.0008	4.94E-01	0.0761	NO
	YKR014C	YNL093W	0.0759	1.87E-01	0.014806	NO
	YFL051C	YKR102W	0.0182	2.43E-01	1	NO
	YGL194C	YNL330C	-0.1576	5.01E-10	0.062676	YES
	YER185W	YGR213C	0.0018	4.74E-01	1	NO
	YDR135C	YLL015W	-0.0374	3.18E-04	0.052111	YES
	YGR152C	YOR101W	-0.0028	4.72E-01	0.01862	NO
	YDR256C	YGR088W	0.0399	1.60E-01	0.089092	NO
	YLR188W	YPL270W	-0.1052	2.48E-14	1	YES
	YLR343W	YMR307W	-0.0382	1.36E-01	0.237288	NO
	YDR525W-A	YJL151C	-0.0057	4.59E-01	1	NO
	YGL257C	YNR059W	-0.0175	3.58E-01	0.17108	NO
	YJR130C	YLL058W	0.0146	3.66E-01	0.670398	NO
	YDR492W	YOL002C	-0.014	4.19E-01	0.012954	NO
	YOL119C	YOR306C	-0.0015	4.80E-01	0.600636	NO
	YGL255W	YLR130C	-0.3464	1.17E-13	0.112086	YES
	YGL058W	YMR022W	0.0243	1.65E-01	0.193171	NO
	YJR032W	YLR216C	0.0519	5.15E-02	0.205504	NO
	YGL002W	YHR110W	0.0076	4.00E-01	0.576299	NO
	YHR030C	YPR054W	-0.024	2.86E-01	0.121446	NO
	YDR258C	YLL026W	-0.042	2.48E-01	0.041073	NO
	YGL125W	YPL023C	-0.017	1.55E-01	1	NO
	YLR262C	YOR089C	0.289	8.98E-06	0.252584	NO
	YDR214W	YNL281W	-0.0347	3.37E-01	0.367768	NO
	YHR104W	YJR096W	0.0307	2.86E-01	0.041073	NO
	YGR121C	YNL142W	0.0042	4.68E-01	0.176699	NO
	YJR058C	YLR170C	-0.0254	8.91E-02	0.422263	NO
	YHR138C	YNL015W	-0.0092	3.81E-01	0.041073	NO
	YIR013C	YLR013W	0.0241	2.59E-01	0.249608	NO
	YLR152C	YNL095C	0.0487	1.66E-01	1	NO
	YMR266W	YOL084W	-0.0305	1.88E-01	1	NO
	YIL107C	YJL155C	-0.0093	4.36E-01	0.301068	NO

	YMR214W	YNL064C	0.0135	3.61E-01	0.070718	NO
	YEL040W	YLR213C	0.0809	5.19E-03	0.242464	NO
	YCR083W	YLR043C	-0.012	3.48E-01	0.54559	NO
	YEL029C	YNR027W	0.0574	1.87E-01	0.058696	NO
	YBR149W	YOR120W	-0.0062	3.46E-01	0.452495	NO
	YBL011W	YKR067W	-0.8523	8.90E-73	0.116411	YES
	YBR015C	YJL186W	-0.0416	3.20E-01	0.003064	NO
	YGR028W	YPL074W	-0.0047	4.34E-01	1	NO
	YER048C	YIR004W	-0.0792	1.49E-01	0.946795	NO
	YGL019W	YOR039W	0.2587	1.98E-08	0.008781	NO
	YKL146W	YNL101W	0.0617	1.54E-03	0.058696	NO
	YBR162C	YJL171C	0.0361	2.15E-01	1	NO
	YOR348C	YPL265W	-0.0333	7.12E-02	0.206631	NO
	YBR291C	YJR095W	-0.0476	7.30E-02	0.370555	NO
	YIL006W	YIL134W	0.0694	2.72E-10	0.289059	NO
	YBL089W	YIL088C	-0.0006	4.34E-01	0.020095	NO
	YMR272C	YNL111C	-0.1269	9.57E-02	0.484822	NO
	YBR267W	YLR387C	-0.4066	4.28E-54	0.048182	YES
	YMR316W	YOR385W	0.0176	2.53E-01	1	NO
	YDL123W	YDR276C	-0.0413	1.39E-01	1	NO
	YLR098C	YOR337W	-0.0105	4.03E-01	0.280795	NO
	YCR106W	YLL054C	-0.0242	3.05E-01	1	NO
	YMR166C	YNL003C	-0.0239	3.96E-01	0.600636	NO
	YER175C	YHR209W	0.0522	9.41E-74	1	NO
	YAL039C	YKL087C	0.053	1.21E-01	0.168488	NO
	YIL130W	YJL206C	-0.03	2.72E-01	1	NO
	YDR387C	YOL103W	0.0393	1.14E-01	0.561502	NO
	YOL027C	YPR125W	-0.0948	6.82E-16	0.149293	YES
	YDR261C	YOR190W	0.0131	4.12E-01	0.948016	NO
	YER072W	YJL012C	0.018	2.70E-01	0.21364	NO
	YJR152W	YLR004C	-0.0311	2.84E-02	0.576807	YES
	YHR028C	YOR219C	0.0145	4.16E-01	0.218474	NO
	YCR021C	YDR033W	0.0508	4.52E-05	1	NO
	YER086W	YKL218C	0.0067	4.02E-01	0.47455	NO
	YAL026C	YDR093W	0.0235	3.38E-01	0.000673	NO
	YMR210W	YPL095C	0.0034	4.72E-01	0.112086	NO
	YBR125C	YDL006W	0.0096	4.31E-01	0.310906	NO
	YIL002C	YOR109W	-0.0156	3.85E-02	0.044516	YES
	YDL127W	YNL289W	-0.0407	3.09E-01	0.026517	NO
	YJL193W	YOR307C	0.0008	4.94E-01	1	NO
	YBR033W	YKL038W	0.0448	1.30E-02	1	NO
	YDL222C	YML052W	0	4.99E-01	0.092024	NO
	YHR049W	YOR280C	-0.2046	8.03E-04	1	YES
	YDR144C	YIL015W	0.0095	2.23E-01	0.596005	NO
	YJL212C	YPR194C	0.0026	4.70E-01	0.245085	NO
	YGR130C	YLR200W	-0.0448	2.98E-02	1	YES

	YBL063W	YEL061C	-0.8641	3.21E-11	0.051246	YES
	YHL019C	YPL259C	0.013	4.19E-01	0.282874	NO
	YKR042W	YMR244W	-0.0339	2.18E-01	1	NO
	YDL229W	YHR064C	-0.0323	2.23E-03	0.079646	YES
	YBR207W	YER145C	0.0125	1.00E-01	0.1522	NO
	YBR105C	YGR066C	-0.0049	4.21E-01	1	NO
	YGL202W	YHR137W	-0.001	4.83E-01	0.38027	NO
	YFL018C	YPL091W	-0.0842	1.29E-01	1	NO
	YDR191W	YOR025W	-0.7155	0.00E+00	0.062676	YES
	YGR055W	YHL036W	0.0121	2.06E-01	0.283686	NO
	YDR148C	YNL071W	-0.153	5.44E-02	0.57662	NO
	YDL230W	YOR208W	-0.0092	4.11E-01	0.218653	NO
	YJR116W	YPR114W	0.0343	2.80E-02	1	NO
	YDR435C	YOL141W	-0.0127	4.05E-01	0.408067	NO
	YJL036W	YOR357C	-0.2351	1.44E-03	0.112086	YES
	YCR045C	YEL060C	-0.002	4.88E-01	0.316112	NO
	YBR028C	YCR091W	-0.0171	2.19E-01	1	NO
	YGR203W	YPR200C	0.1358	1.04E-01	1	NO
	YIL079C	YNL255C	0.0338	2.13E-01	0.684506	NO
	YCL001W-A	YNL001W	0.0317	1.87E-01	1	NO
	YAR002C-A	YGL200C	-0.0031	4.42E-01	0.234415	NO
	YMR159C	YOR195W	-0.0681	4.57E-02	0.690349	YES
	YDR244W	YMR018W	0.0834	6.79E-02	1	NO
	YBL069W	YMR152W	0.0061	4.29E-01	1	NO
	YBR073W	YGL163C	-0.0764	7.98E-06	0.175425	YES
	YBR293W	YMR088C	-0.0394	3.13E-01	0.300809	NO
	YDR352W	YOL092W	-0.0504	2.05E-01	1	NO
	YIL153W	YPL152W	-0.7338	0.00E+00	0.198532	YES
	YDL168W	YLR070C	0.0281	2.80E-01	0.459979	NO
	YAL021C	YOL042W	-0.0158	2.47E-01	0.497424	NO
	YDL025C	YNL183C	0.039	4.68E-02	0.642623	NO
	YHL039W	YPL208W	0.0071	4.37E-01	1	NO
	YDR131C	YJL149W	-0.0057	3.99E-01	0.204799	NO
	YGR232W	YOR034C	-0.0834	1.04E-01	0.598838	NO
	YLR090W	YNL077W	0.0042	3.32E-01	1	NO
	YFR021W	YPL100W	-0.0931	6.07E-02	0.132855	NO
	YBR212W	YHR086W	-0.1042	9.11E-02	0.662198	NO
	YKR035W-A	YLR309C	0.0135	2.18E-01	0.31279	NO
	YDR097C	YHR120W	0.009	2.07E-01	0.043681	NO
	YLR214W	YNR060W	0.0034	4.74E-01	0.195843	NO
	YER166W	YMR162C	-0.0204	2.52E-01	0.000673	NO
	YER113C	YLR083C	0.0093	2.30E-01	0.182804	NO
	YNL020C	YPL236C	-0.0833	1.16E-01	0.760066	NO
	YGL059W	YIL042C	-0.0354	1.67E-01	0.219819	NO
	YJL070C	YML035C	-0.1678	1.43E-26	1	YES
	YGR068C	YOR018W	-0.0028	4.73E-01	0.410687	NO

	YCR009C	YDR388W	0.0832	1.06E-11	0.014806	NO
	YGR282C	YMR305C	0.0181	2.41E-01	0.096775	NO
	YDL113C	YDR425W	-0.0089	3.32E-01	0.06794	NO
	YDR192C	YKL068W	-0.0688	2.40E-01	0.022256	NO
	YAL007C	YDL018C	0.0337	2.32E-01	0.576299	NO
	YBR180W	YNL065W	-0.0379	9.10E-02	0.448979	NO
	YBR168W	YLR324W	-0.0182	3.97E-01	0.578441	NO
	YDL099W	YNR010W	-0.0306	2.34E-01	0.934353	NO
	YGL160W	YLR047C	-0.0351	2.89E-01	1	NO
	YFL040W	YHR092C	0.0244	1.85E-02	0.522103	NO
	YBR058C	YMR223W	0.0439	1.25E-01	0.171311	NO
	YIL149C	YLR211C	-0.0238	3.55E-01	1	NO
	YGL094C	YOL080C	0.0056	4.04E-01	0.240116	NO
	YCL040W	YLR446W	-0.0102	1.59E-01	1	NO
	YPL159C	YPR151C	0.0033	4.76E-01	0.538414	NO
	YDR293C	YPR101W	-0.1509	1.58E-04	0.932193	YES
	YFL023W	YPL186C	-0.0131	2.97E-01	1	NO
	YLR251W	YOR292C	-0.0029	4.61E-01	1	NO
	YNR009W	YPR171W	0.0294	7.12E-02	0.747121	NO
	YLR401C	YNR015W	-0.0152	2.94E-01	0.060673	NO
	YGL080W	YHR162W	-0.0021	4.80E-01	1	NO
	YDR334W	YJR035W	0.0213	6.14E-03	0.919641	NO
	YBL064C	YML028W	-0.0599	4.28E-02	0.48889	YES
	YBR274W	YGL180W	-0.0437	3.97E-01	0.121446	NO
	YJR005W	YKL135C	-0.0444	1.86E-01	0.422263	NO
	YCR011C	YKL061W	-0.0226	2.41E-01	1	NO
	YKL023W	YLR068W	-0.013	2.90E-01	1	NO
	YML096W	YPR145W	-0.0088	4.05E-01	1	NO
	YKL079W	YPL155C	0.0053	2.80E-01	0.516244	NO
	YER107C	YOR026W	-0.136	7.72E-04	0.387207	YES
	YCR008W	YJL165C	-0.8397	3.26E-127	0.043105	YES
	YBL005W	YER184C	0.0322	5.52E-02	1	NO
	YKL188C	YPL147W	0.0197	3.47E-01	0.139232	NO
	YDL065C	YNL136W	-0.1042	3.80E-02	0.503805	YES
	YDL197C	YDR285W	-0.0188	3.48E-01	0.941301	NO
	YBR295W	YDR270W	-0.0319	3.38E-01	0.022564	NO
	YNL175C	YOL041C	-0.0674	2.31E-01	1	NO
	YDR067C	YNL032W	0.0421	1.82E-01	1	NO
	YDL154W	YOL090W	-0.0093	4.37E-01	0.249203	NO
	YER090W	YNR033W	0.0682	9.00E-02	0.320286	NO
	YMR105C	YMR278W	-0.0081	2.69E-01	1	NO
	YDR406W	YOL075C	0.0394	2.04E-01	0.020095	NO
	YDR074W	YMR261C	0.0083	5.50E-03	0.182804	NO
	YER151C	YHR159W	-0.0305	3.05E-01	1	NO
	YAL042W	YML067C	0.0362	6.53E-11	0.234415	NO
